# Food-relevant functional properties of the four major bovine casein fractions: A review

**DOI:** 10.1016/j.crfs.2026.101618

**Published:** 2026-11-02

**Authors:** Dana Chezan, Philipp L. Fuhrmann, Konrad J. Domig, Belinda P.C. Dewi

**Affiliations:** a BOKU University, Department of Biotechnology and Food Science, Institute of Food Science, Muthgasse 18, 1190, Vienna, Austria; b FFoQSI– Austrian Competence Centre for Feed and Food Quality, Safety & Innovation, Head Office: FFoQSI GmbH, Technopark 1D, 3430, Tulln, Austria

**Keywords:** Casein fractions, Ingredient functionality, Physicochemical properties, Dairy functionality, Casein micelle, Dairy proteins

## Abstract

This review critically evaluates the functional properties of the primary bovine casein fractions (α_s1_-, α_s2_-, β- and κ-caseins) that are relevant to food systems, focusing on their physicochemical characteristics. It also considers the genetic variation and post-translational modifications (PTMs) among these fractions, which directly affect their properties and functionalities. The gelation of individual casein fractions is primarily influenced by their isoelectric points (pI) and protein-protein interactions. β-casein has an amphiphilic and flexible structure, contributing to its versatility in terms of foaming and emulsifying capabilities. In contrast, α_s1_-casein has strong calcium-binding properties, and κ-casein plays an important role in steric stabilization at interfaces, particularly in maintaining micelle stability through its glycosylated C-terminal (caseinmacropeptide, CMP) region. Technological challenges in isolating pure casein fractions may contribute to a significant knowledge gap due to the scarcity of systematic studies using purified fractions; β-casein has been studied more extensively than the other casein fractions. Consequently, comprehensive conclusions about the functionalities of α_s1_-, α_s2_-, and κ-caseins remain limited. Furthermore, genetic variants were not consistently reported in studies on isolated casein fractions, despite evidence suggesting that these variants influence functional properties. Emerging technologies like precision fermentation and advanced fractionation techniques are expected to enable more targeted functional applications in casein-based ingredients. Therefore, a systematic characterization of casein genetic variants is crucial for optimizing their application in tailored dairy product formulation. Further research in real food matrices, such as dairy systems, is necessary to accurately assess and optimize the techno-functional properties of individual casein fractions.

## Highlights

• β-casein has been studied more extensively than α_s1_-, α_s2_-, and κ-casein.
• Each casein fraction has distinct functional properties relevant for food applications.
• Minor genetic variability in casein fractions affects their functional properties.
• Casein fractions' physicochemical properties are strongly influenced by environmental conditions.

## Graphical abstract

**Figure:**
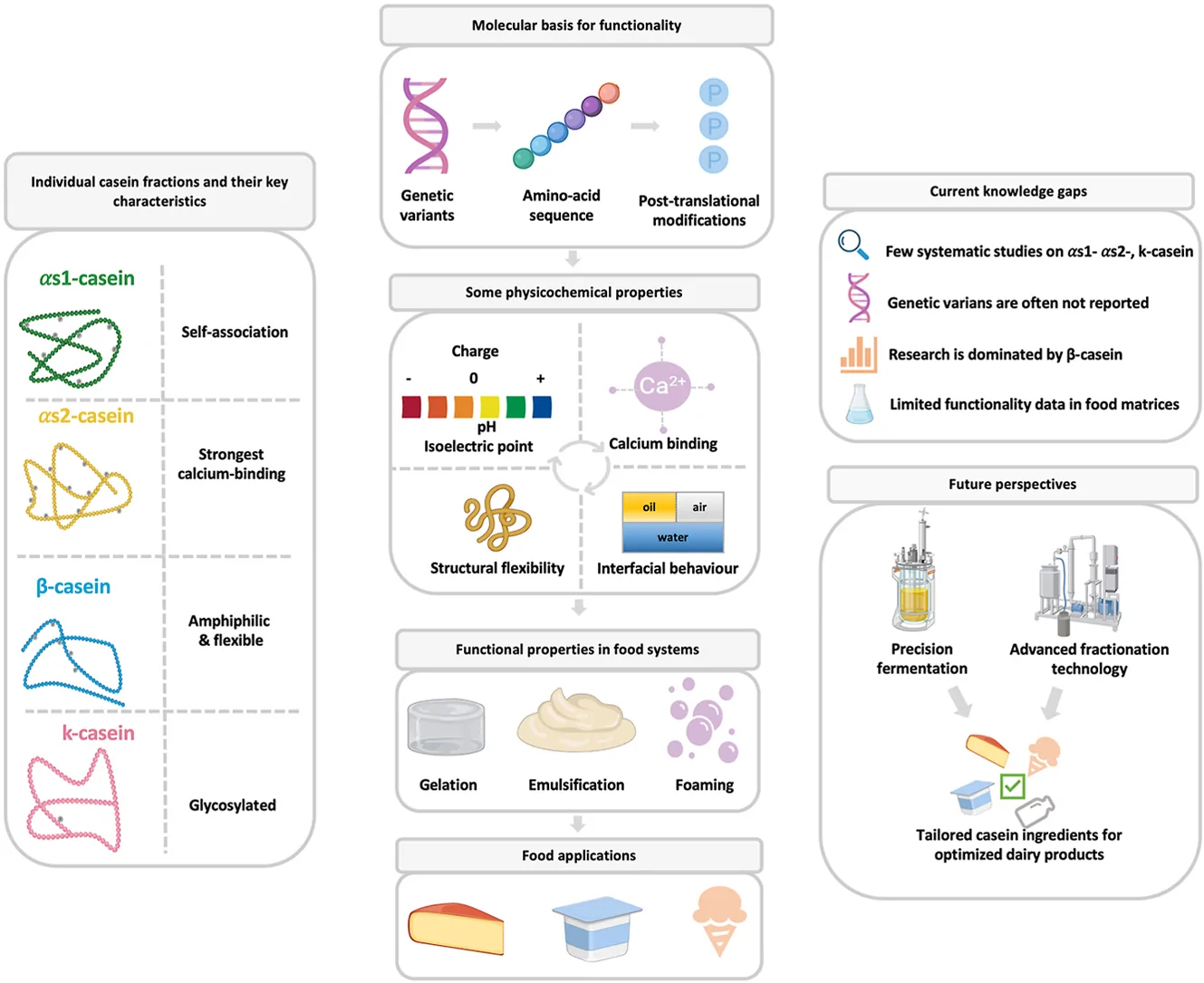

## Abbreviations

BSA -: Bovine Serum Albumin
CMP -: Casein Macropeptide
BLG -: β-Lactoglobulin
WPI -: Whey Protein Isolate
PTM(s) –: Post-translational modifications

## 1 Introduction

Bovine milk is an important food and ingredient, valued for its rich nutritional profile, which includes proteins, fats, lactose and minerals. Throughout this review, milk is referred to as bovine milk, except where it is mentioned differently. The milk proteins, in particular, are crucial for the long-known functional properties of milk such as foaming, emulsification and gelation (Roy et al., 2020). The two major protein fractions in milk are caseins and whey proteins, occurring at an approximate 80:20 ratio (Haug et al., 2007; Toffolon et al., 2021). A more detailed schematic representation of milk composition is shown in Fig. 1. Caseins contribute to the structure and stability of a range of foods, including cheese, yoghurt, whipped cream, and milk-based foams (Besner and Kessler, 1998; Li et al., 2020, Li et al., 2020; O'Kennedy, 2011; Zhao et al., 2008). They consist of four main fractions, α_s1_-, α_s2_-, β-, and κ-caseins, present in approximate proportions of 40%, 10%, 35%, and 15%, respectively. In their native form, these fractions are structured as a colloidal particle, known as the casein micelle (cf. Fig. 1). These micellar structures are stabilized by colloidal calcium phosphate (CCP) and a κ-casein-rich surface layer. Fig. 1 also shows the structural hierarchy of caseins, from micelles to dissociated caseinates and individual casein fractions, and how they can be hydrolyzed into smaller peptides.

**Fig. 1:**
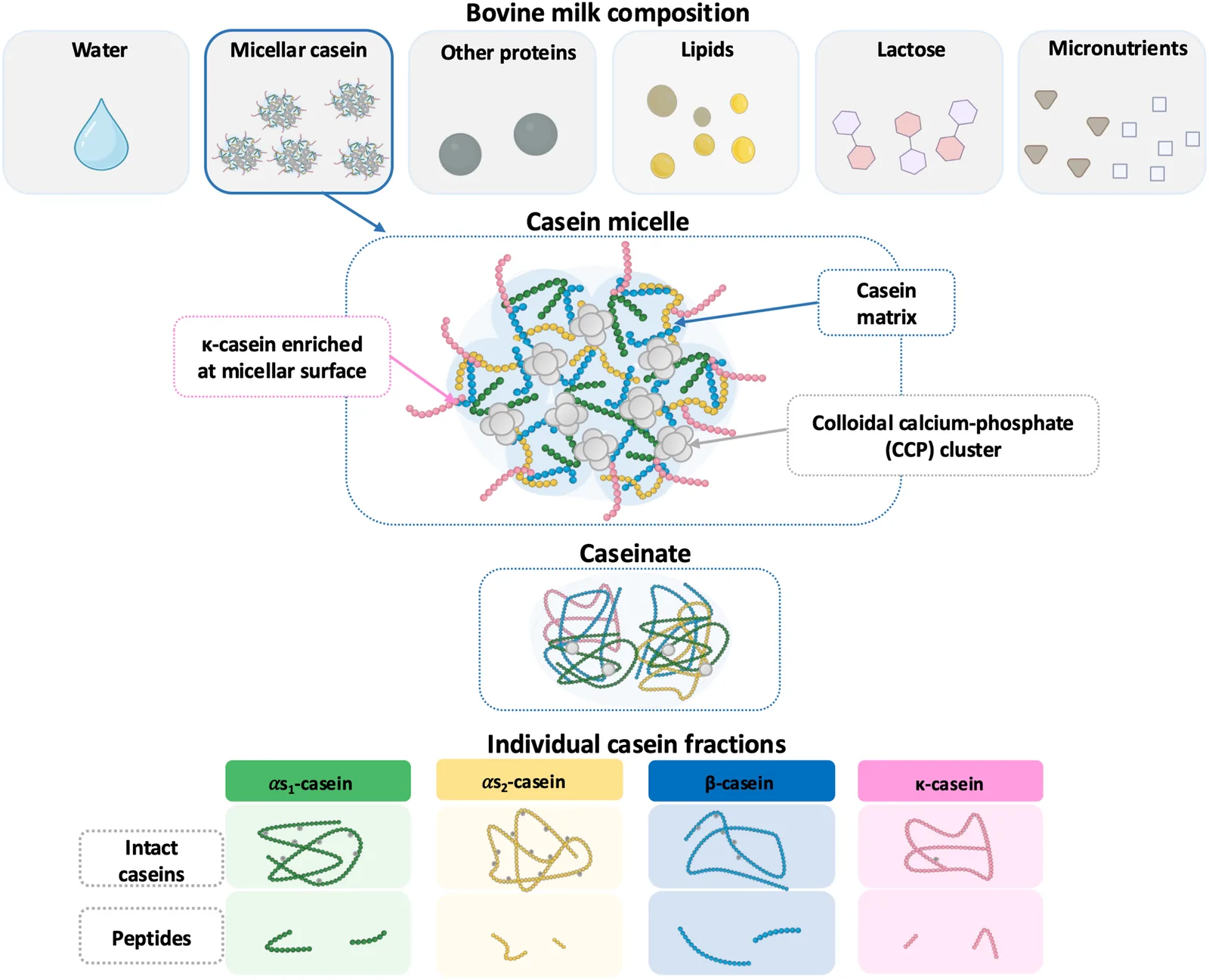
Schematic overview of bovine milk composition, casein micelles, caseinate and individual casein fractions**.** The simplified micelle representation reflects the model of Huppertz et al. (2017). A recent review summarized that the precise internal organization of native micelles remains unresolved (Anema, 2026). Green, yellow, blue and pink depict α_s1_-, α_s2_-, β- and κ-casein, respectively. Intact caseins and schematic peptide fragments that may be produced by hydrolysis are shown.

Despite decades of research dedicated to understanding the structure and function of casein micelles, surprisingly, little is known about the functional properties of the individual casein fractions. This lack of detailed characterization is becoming increasingly significant as standardizing milk is gaining importance for ensuring consistent composition, optimizing processing efficiency, and maximizing yield in dairy industry applications (Guinee et al., 2006; Kalit et al., 2021; Rattray and Jelen, 1996). In addition to overall milk standardization, variation in the relative proportions of individual casein fractions can also affect the functional properties of dairy products. For example, higher β-casein content has been associated with greater cheese yield, while higher κ-casein and α_s1_-casein levels were linked to improved retention of cheese solids (Cipolat-Gotet et al., 2018). Furthermore, genetic polymorphisms are another important source of variation in casein functionality. There is substantial evidence of the effects on functional properties in whole milk and casein-derived ingredients (Bittante et al., 2012; Daniloski et al., 2022; Gai et al., 2021; Hewa Nadugala et al., 2022, 2024, 2025; Ho et al., 2022; Huppertz, 2010; Ketto et al., 2018, 2019) as well as in more complex dairy systems (Gai et al., 2024; Logan et al., 2027; Nguyen et al., 2018; Ren et al., 2013). For example, Ren et al. (2013) showed that κ-casein allelic variants play an important role in the functional properties of mozzarella cheese. The AB genotype had the most favorable effect on the stretchiness, chewiness and hardness of the final product.

Studies using isolated casein fractions from homozygous cows have also examined β-casein variants A1, A2, B and I (Petrat-Melin et al., 2015) and κ-casein variants A, B and E (Sheng et al., 2022), although their focus was on in vitro digestion rather than the techno-functional properties considered here. Despite the demonstrated importance of casein composition and genetic variation, their effects on the techno-functional properties of isolated casein fractions remain comparatively understudied. Challenges in obtaining individual casein fractions with sufficient purity and recovery may contribute to their limited functional characterization. For example, food-grade αs-casein with purities below 65% relative to total casein was obtained in technical- and pilot-scale fractionation studies (Atamer et al., 2017a, Atamer et al., 2017b; Thienel et al., 2018). Although α_s1_-and α_s2_-caseins are distinct proteins, they are often referred to collectively as αs-casein in the literature. β-casein fractions have been obtained with purities of over 90% and yields of up to 32% relative to the total starting protein under different processing conditions. This may have facilitated more detailed investigations into its functional and structural properties (Atamer et al., 2017a, Atamer et al., 2017b; Post et al., 2009; Post and Hinrichs, 2010, 2011; Schubert et al., 2021; Thienel et al., 2018; van der Schaaf et al., 2024). The existing insights on κ-casein focus primarily on its unique structural role in micelle stabilization, genetic polymorphisms and its derived peptide, the CMP (Creamer et al., 1998; Manguy and Shields, 2019; Ninan and Vadodaria, 2024). At pilot scale, Schubert et al. (2018) obtained a κ-casein-enriched fraction with approximately 54% purity relative to total casein and 83% fraction-based recovery. Higher purities of 90.0-97.1% were achieved for κ-casein variants A, B, and E at laboratory scale using reducing anion-exchange chromatography, with reported yields ranging from 9% to 29% (Sheng et al., 2022). These findings highlight the importance of considering purity and recovery alongside processing conditions and scale in κ-casein fractionation.

Alongside advances in casein fractionation, recent developments in food biotechnology, particularly precision fermentation, have made the separate production of casein fractions increasingly feasible (Chezan et al., 2026; Knychala et al., 2024; Mortes et al., 2026; Nielsen et al., 2024; Wang et al., 2021, 2026). Recombinant technology enables the production of casein fractions with amino acid sequences comparable to those of native bovine caseins. The process involves introducing the gene encoding the target protein into a suitable host, i.e., bacteria, yeast or fungi. Following protein production through fermentation (upstream processing), the target protein is recovered and purified through downstream processing. The choice of production host and the upstream and downstream processing conditions can influence protein yield and quality. Thus, these steps are an active area of research (Iaria et al., 2026; Piazenski et al., 2024). These scientific and technological advances have also attracted industrial interest, with several companies developing recombinant proteins for food and animal feed applications (Graham and Ledesma-Amaro, 2023). Together with advances in casein fractionation, these developments make individual casein fractions increasingly accessible. Therefore, consolidating the existing knowledge on their functional properties is increasingly relevant. In this review, we critically examine the existing literature on the physicochemical and food-relevant functional properties of individual casein fractions. First, we focus on their physicochemical properties, followed by their main food-relevant functionalities: gelation, emulsification and foaming. Finally, interactions between casein fractions and other food components, such as polysaccharides, are discussed. Throughout this review, the focus is on individual, isolated casein fractions. Studies on caseins in whole milk, native casein micelles, micellar casein preparations, caseinates or unfractionated casein mixtures are outside our scope. Nutritional, digestive and physiological properties are also outside our scope. Where direct comparisons among all four purified fractions are unavailable, studies using other proteins may occasionally be used as comparators to provide functional context.

## 2 Casein fractions characterization

In this section, the first part focuses on basic aspects involving protein characterization, such as size, PTMs, and structure, followed by solubility, aggregation behavior, and calcium sensitivity, which form the basis for understanding the functional properties of casein fractions in food applications.

### 2.1 Genetic polymorphism, size and charge

Bovine casein has at least 39 genetic variants, which are distributed among the different casein fractions as follows: 8 for α_s1_-casein, 4 for α_s2_-casein, 15 for β-casein and 12 for κ-casein (Daniloski et al., 2022a, Daniloski et al., 2022b). Common variants include α_s1_-(B variant), α_s2_-(A variant), β-(A2 variant), and κ-caseins (B variant), respectively (Broyard and Gaucheron, 2015). The molecular masses of all four casein fractions in these various genetic variants range from approximately 19.0 to 25.2 kDa (Broyard and Gaucheron, 2015, Table 1).

**Table 1:** General characteristics of casein fractions^a^.

Casein fraction (genetic variant)	Number of amino acids (n)	Proline content (%)	Hydrophobicity index (%)	Phosphoryla-tion sites (n)	Glycosylation sites (n)	Notes
αs1-casein (B)	199	8.5	43.9	8	0	Intermediate structure flexibility
αs2-casein (A)	207	4.8	31.7	11	0	Most basic residues
β-casein (A2)	209	16.7	51.6	5	0	Most hydrophobic, flexible
κ-casein (B)	169	11.8	45.5	1	0-5	Provides steric stabilization of micelles

a Modified from (Broyard and Gaucheron, 2015; Hassanin et al., 2022; Petrova et al., 2022).

Genetic variants result from differences in the DNA sequences encoding the individual caseins, leading to differences in their amino acid sequences. In addition to genetic variation, caseins differ in their PTMs, particularly phosphorylation and glycosylation. Glycosylation involves the attachment of carbohydrate groups. Among the major bovine casein fractions, glycosylation occurs in κ-casein (Bonfatti et al., 2014). Meanwhile, phosphorylation involves the addition of phosphate groups, mainly to serine residues. The negatively charged phosphate groups contribute to the charge of caseins and their ability to bind calcium. The negatively charged phosphate groups increase the net negative charge of casein molecules (Broyard and Gaucheron, 2015). For example, as shown in the work of Chezan et al. (2026), native bovine β-casein with five phosphate groups had a more negative zeta potential compared to non-phosphorylated β-casein derived from precision fermentation. Thus, both phosphorylation and glycosylation significantly alter the structural and physicochemical characteristics of caseins, influencing key functional properties such as calcium sensitivity, aggregation behavior, solubility, and molecular structure (Broyard and Gaucheron, 2015; Darewicz et al., 1998, 2000; McCarthy et al., 2013; Molina et al., 2007). These are all critical to their behavior in dairy systems and will be discussed in more detail in the following sections.

### 2.2 Primary, secondary, and higher order organization

The four main bovine casein fractions, α_s1_-, α_s2_-, β- and κ-casein in their common genetic variants (B, A, A2, and B) considered here are distinguished by their amino acid composition and structural characteristics. α_s1_-casein consists of 199 amino acids, of which 17 are prolines (8.5%), with a cluster of phosphoserines located between positions 41 and 80. α_s2_-casein consists of 207 amino acids, contains 10 prolines (4.8%) and has a basic C-terminal region. β-casein (A2) contains 209 amino acids, making it the most proline-rich (approximately 16.7%). It is characterized by a highly amphiphilic nature and a hydrophobic C-terminal region. κ-casein (B) contains 169 amino acids and includes two cysteines (Cys11 and Cys88), which enable the formation of disulfide bonds. Table 1 provides further information on the common genetic variants of the four casein fractions (Broyard and Gaucheron, 2015).

Based on their primary sequences, the β- and α_s1_-casein fractions are considered intrinsically disordered proteins (IDPs), characterized by a high proline content which disrupts organized secondary motif content and the absence of cysteine residues, which limits the formation of stabilizing disulfide bonds. Although κ-casein and α_s2_-casein contain cysteine residues, they are also classified as IDPs due to their overall disordered structure and lack of stable tertiary conformation. Typically, IDPs do not have a fixed structure but instead exist as dynamic conformational ensembles, which may retain limited and transient secondary structural elements (Carver and Holt, 2019; Holt et al., 2013). Based on the literature included in this review, Grewal et al. (2021) is the only study comparing the secondary structures of all four highly purified (>95%) bovine casein fractions using Fourier-transform infrared (FTIR) spectroscopy under identical experimental conditions. The four fractions showed distinct proportions of ordered secondary structural elements (α-helices, β-sheets, 3_10_-helices and β-turns) at 25 °C in a 25 mM PIPES buffer solution containing 100 mM KCl. α_s1_-and α_s2_-caseins contained the highest proportions of β-sheet structures, while β-casein displayed the lowest β-sheet content and the highest proportion of random coil structures. This is consistent with its greater conformational flexibility. Adding Ca^2+^ or Mg^2+^ altered the secondary structure of all the casein fractions, generally promoting β-sheet formation while decreasing 3_10_-helices. The response was casein-specific, depending on the type and concentration of the divalent cation present (Grewal et al., 2021). By contrast, previous studies have mostly focused on either individual casein fractions or selected subsets, rather than comparing all four fractions systematically. Furthermore, reported secondary structure values vary considerably because of differences in analytical techniques (e.g. sequence-based prediction, circular dichroism (CD), Raman spectroscopy, FTIR spectroscopy), experimental conditions, and the purity and genetic variants of the caseins studied (Swaisgood, 1993). Even under identical experimental conditions, estimated secondary structure values may vary depending on the analytical method used (Creamer et al., 1981). Accordingly, emphasis is placed on overall structural trends rather than exact numerical values. Based on CD measurements and under near-neutral conditions (pH 6.8), α_s1_-casein was predominantly disordered, with random coil structures accounting for roughly 60–70% of its conformation. Acidification induced a partial shift toward more ordered structures, with α-helical and β-sheet contents increasing from 13 to 20% to approximately 26-27% and from 17% to 24%, respectively, alongside a reduction in disordered structure to about 50%. A similar trend was observed for β-casein. However, it remained largely disordered, retaining 68% random coil content and exhibiting minimal β-sheet formation even under acidic conditions. These findings indicate that while acidic environments promote partial structural ordering, intrinsic disorder remains a defining characteristic of casein proteins (Creamer et al., 1981). The secondary structure of β-casein was also affected by temperature. At 10 °C in a 50 mM sodium phosphate buffer solution (pH 7.4), β-casein consisted of approximately 10% α-helix, 12% β-strand, 14% turn and 64% unordered structure as determined by CD spectroscopy. Increasing the temperature to 50 °C resulted in an increase in ordered elements and a decrease in disorder, indicating thermally induced structural reorganization (Faizullin et al., 2017). Such changes in casein conformation may alter intermolecular interactions and thereby influence subsequent association and aggregation behavior. These findings are consistent with the work of other authors (Markoska et al., 2021; Zhang et al., 2024). A notable feature of β-casein is that its secondary structure remained relatively unchanged between 20 °C and 4 °C at pH 5.9. This stability at pH 5.9 is likely due to stronger hydrophobic interactions and reduced electrostatic repulsion, which limit conformational changes at lower temperatures. In contrast, a significant structural rearrangement occurred at pH 7.0 (Markoska et al., 2021). Furthermore, an increase in the strength of the sodium phosphate buffer from 10 to 50 mM resulted in an increase in the overall ordered secondary structures of β-casein, including both α-helices and β-sheets (Chezan et al., 2026). Interactions with other compounds can also affect the conformation of β-casein, as demonstrated by the binding of tetracycline hydrochloride (TCH) (Bi et al., 2016). Intramolecular crosslinking has further provided evidence of the conformational flexibility of β-casein (Livney et al., 2004). κ-casein had a stable, partially β-sheet conformation in a 0.01 M imidazole buffer at pH 7.0 and an ionic strength of 0.07, as indicated by CD spectroscopy at 215 nm. Despite its high proline content, the structure remained relatively rigid and calcium binding did not affect it (Ono and Odagiri, 1980). Kumosinski et al. (1991) proposed a three-dimensional model of κ-casein with a “horse and rider” configuration, stabilized by β-sheets and disulfide bonds between cysteines. In conclusion, although all four casein fractions are intrinsically disordered, they differ in their secondary structure profiles and respond to environmental conditions such as pH, temperature and ionic composition. Under comparable experimental conditions, α_s1_-and α_s2_-caseins show a greater proportion of ordered secondary structural elements, whereas β-casein displays a higher degree of disorder and conformational flexibility. κ-casein also remains intrinsically disordered but contains partially ordered structural elements and appears less conformationally flexible than β-casein. Overall, these findings highlight both fraction-specific structural differences and the influence of the surrounding environment on casein conformation.

### 2.3 Zeta potential

Zeta potential reflects the surface charge of proteins and provides information on electrostatic interactions that can influence their colloidal behavior in solution. These charge-dependent differences are therefore particularly relevant to the aggregation and solubility behavior discussed in the following sections. In the context of individual casein fractions, however, limited data are available. To our knowledge, no studies have specifically reported the zeta potential of isolated α_s2_-casein, possibly reflecting, in part, the difficulty of obtaining this fraction at high purity. Rodzik et al. (2020) measured the zeta potential of the major casein fractions (α_s1_-, β-, and κ-casein) across pH 2-11 in 0.09% NaCl. Readings were taken at 1 min, 1 h, and 5 h. For α_s1_-casein, the pI was approximately 4.80 ± 0.72. At low pH, it had a positive zeta potential, which became increasingly negative above the pI, reaching approximately −20 to −30 mV. However, its zeta potential showed greater variation over time than that of β- and κ-casein under the same conditions. In food-grade αs-casein preparations with an approximate purity of 54%, Kessler et al. (2013) found that the composition of the buffer solution affected the zeta potential. They reported values of −18 ± 2 mV for non-dialyzed samples and −23 ± 5 mV for dialyzed ones at pH 6.6 in a 20 mM imidazole buffer solution. These findings further demonstrate that the measured zeta potential depends on the composition of the surrounding medium. β-casein had a pI of 4.55 ± 2.15, with zeta potentials ranging from +32 to −35 mV across the pH range and stabilizing around −20 to −30 mV at pH 5. Its zeta potential also showed less variation over time than that of α_s1_-casein under the same conditions (Rodzik et al., 2020). Similarly, Lajnaf et al., 2021a, Lajnaf et al., 2021b reported increasingly negative zeta potentials for purified bovine β-casein with increasing pH, from −20.9 ± 1.9 mV at pH 5 to −30.0 ± 1.5 mV at pH 7 and −32.9 ± 1.1 mV at pH 9 in 20 mM Tris-HCl buffer. Chezan et al. (2026) further showed that buffer composition and degree of phosphorylation affect the protein's charge. Native β-casein in a 50 mM sodium phosphate buffer at pH 7 had a zeta potential of −23.55 ± 0.54 mV, compared with values below −30 mV reported in demineralized water (Post et al., 2012). Under the same phosphate-buffer conditions, recombinant β-casein measured +12.94 ± 0.05 mV, demonstrating a substantial difference between the native phosphorylated and recombinant non-phosphorylated proteins (Chezan et al., 2026). Overall, the studies show that the zeta potential of individual casein fractions is strongly dependent on pH and solution composition, while differences in protein characteristics, including phosphorylation state, may further influence the measured zeta potential. Direct comparisons of the individual fractions under equivalent conditions remain scarce, and data for isolated α_s2_-casein are particularly lacking.

### 2.4 Aggregation behavior and micelle formation

The precise structure and internal organization of native casein micelles remain a matter of debate, despite decades of extensive research and the proposal of numerous structural models (Anema, 2026; Bittante et al., 2012; De Kruif et al., 2012; Marchin et al., 2007; McMahon and Oommen, 2008). Most of these models broadly support an important structural role for casein-associated calcium phosphate nanoclusters (Bouchoux et al., 2010; De Kruif et al., 2012; Holt, 2021; Marchin et al., 2007; McMahon and Oommen, 2008) together with κ-casein at the micellar surface (Bouchoux et al., 2010; De Kruif and Zhulina, 1996). As no single structural model is currently definitive (Anema, 2026), Fig. 1 presents a simplified schematic based on Huppertz et al. (2017). In this review, we focus on the aggregation and micellization behavior of the individual, isolated casein fractions. In this context, self-association behavior provides a basis for comparing fraction-specific behavior. The critical micelle concentration (CMC) is the concentration above which amphiphilic molecules begin to self-associate into micellar or other supramolecular aggregates. Kessler et al. (2013) showed that the CMC of αs-casein is strongly influenced by the ionic environment, increasing from ∼0.21% in the undialysed sample to ∼0.47% after dialysis. This indicates that ionic interactions play an important role in αs-casein self-association, with reduced ionic strength requiring higher protein concentrations for micelle formation. However, because the preparation contained only approximately 54% αs-casein, these values cannot be considered fraction-specific CMC values for purified α_s1_-or α_s2_-casein. Nevertheless, the observed trend indicates an effect of the ionic environment on αs-casein-containing preparations (Kessler et al., 2013). Typically, β-casein exists as monomers at sufficiently low concentrations, but self-associates above its condition-dependent CMC. Structurally, multiple authors found that β-casein micelles are similar to detergent-like micelles, with a hydrophobic core surrounded by a hydrophilic surface (Aschi et al., 2009; de Kruif and Grinberg, 2002; McCarthy et al., 2013). This is consistent with the model originally proposed by Creamer (1975), where the 20-amino-acid stretch at the C-terminus plays a pivotal role in promoting β-casein aggregation. The CMC of β-caseins is not fixed, but varies significantly with environmental factors like temperature, pH, ionic strength and the presence of co-solutes. For β-casein, a CMC of 0.05% was reported by O'Connell et al. (2003) at 40 °C in 25 mM sodium phosphate buffer and pH 6.5. A value in the same range (0.07%) was reported in phosphate-buffered saline (PBS) at pH 7.0, 25 °C and an ionic strength of 0.1 M (Setter and Livney, 2015). Furthermore, in a phosphate buffer solution at pH 7.0 and an ionic strength of 0.1M, the CMC decreased from 0.15 mM (0.36%) to 0.006 mM (0.014%) as the temperature increased from 16 °C to 45 °C (Portnaya et al., 2006). Moitzi et al. (2008) demonstrated that, at pH values above 5.3, temperature was the dominant factor influencing β-casein aggregation, outweighing the effects of pH, protein concentration, ionic strength and solvent composition. The decrease in critical micelle concentration with increasing temperature indicates that progressively lower β-casein concentrations are required for self-association. This behavior is consistent with an increasing contribution of hydrophobic interactions at higher temperatures. Zhang et al. (2024) suggested that β-casein micellization is influenced not only by hydrophobic interactions but also by electrostatic interactions and hydrogen bonding. Increasing pH may weaken these interactions through deprotonation of ionizable side chains, thereby reducing the tendency of β-casein to self-associate. Acidic conditions (pH 2.6) still supported β-casein micellization, but produced disk-like micelles (approximately 20-25 nm wide and 3.5 nm thick) with low aggregation numbers (around six monomers) and a higher CMC (Portnaya et al., 2008). Sugars and other co-solutes can further lower the CMC, and the effect depends on the specific sugar's stereochemistry (Setter and Livney, 2015). Despite variations in experimental setups, studies have reported comparable CMC values and consistently observed temperature-dependent behavior. In addition to environmental factors, the genetic variant of β-casein affects its self association. The A1 variant showed a greater tendency to self associate than the A2 variant under the conditions investigated. Both variants formed micelles measuring between 20 and 25 nm, as demonstrated by dynamic light scattering (DLS). At 0.4% and at temperatures between 20 and 70 °C in a 50 mM sodium phosphate buffer at pH 7, A1 β-casein consistently formed larger micelles than A2 β-casein (Raynes et al., 2015). Overall, the self-association of β-casein is highly condition-dependent, with temperature being one of the most consistently reported determinants, largely through its effect on hydrophobic interactions. While most studies have focused on β-casein, a small number have also examined κ-casein. κ-casein formed different aggregate sizes, depending on the conditions and techniques used. Alkaline polyacrylamide gel electrophoresis (PAGE) of size-exclusion-purified κ-casein (B) showed stable oligomers of at least 250 kDa, while sodium dodecyl sulfate (SDS)-PAGE under non-reducing conditions detected monomers in unheated samples, which were likely held together by non-covalent hydrophobic interactions. These disappeared when the sample was heated to 80 °C, consistent with heat-induced aggregation (Cho et al., 2003). In contrast, light scattering and ultracentrifugation detected much larger aggregates (≈630 kDa) (Farrell et al., 1999), reflecting dynamic higher-order assemblies. This discrepancy in molecular weight may be due to the different methods used. PAGE primarily detects stable or covalently linked aggregates, whereas scattering techniques capture a wider range of transient, non-covalent assemblies. Furthermore, the aggregation behavior of κ-casein is influenced by pH, as demonstrated by Risso et al. (2008), which affects the balance between monomeric and aggregated states. Under reducing conditions at pH 7.2 and 30 °C (concentration ≤0.4%), κ-casein remained predominantly monomeric. Under acidic conditions (pH 5.2), aggregation increased, and mild heating to 40 °C further promoted the formation of larger aggregates. The detection of monomers only under reducing conditions suggests that disulfide bonds are a key factor in aggregation. However, the enhanced aggregation at low pH, where disulfide bond formation is less favorable, suggests that hydrophobic interactions also play a role. This is supported by the increased surface hydrophobicity observed in acidic samples (Risso et al., 2008). Another interesting feature of purified κ-casein is the ability to form fibrils, which are elongated, thread-like aggregates that can form at near-neutral pH and approximately 37 °C. Unlike the native spherical micelles found in milk, these fibrillar aggregates represent an alternative assembly pathway for κ-casein. Under reducing conditions that disrupt disulfide bonds, κ-casein formed amyloid-like fibrils more readily, as confirmed by transmission electron microscopy (TEM) and X-ray diffraction. Fibril formation can also occur, albeit less efficiently, in the absence of reducing conditions (Ossowski et al., 2012). Glycosylation can further influence this self-assembly. Hewa Nadugala et al. (2023) found similar initial aggregate sizes for unglycosylated and twice-glycosylated κ-casein B, but the glycosylated fraction formed fibrils at lower concentrations and produced longer, narrower fibrils at 37 °C (Hewa Nadugala et al., 2023). Fibril formation is not unique to κ-casein: αs- and β-caseins can also form amyloid fibrils when incubated at elevated temperatures (e.g. 65 °C for more than 10 days) (Bahraminejad et al., 2022). At the same time, αs- and β-caseins have been reported to exhibit chaperone-like activity that can suppress κ-casein fibril formation by stabilizing soluble intermediates (Ossowski et al., 2012; Raynes et al., 2015; Thorn et al., 2005). Overall, while several individual casein fractions can form fibrillar structures under controlled laboratory conditions, evidence for the relevance of these structures to food functionality remains limited, particularly in complex food matrices.

### 2.5 Calcium binding

The ability of caseins to bind calcium is crucial for both their biological role and their functionality in food. It contributes to the aggregation and structural behavior of the individual casein fractions and, in dairy systems, influences texture, stability and rheological properties. From a biological perspective, calcium binding also contributes to the transport of calcium in milk. Calcium binding in caseins occurs predominantly through negatively charged phosphoserine residues introduced through post-translational phosphorylation, particularly when these residues occur in phosphorylated clusters, while carboxylate groups can also participate in binding (Aoki et al., 1985; Farrell, Jr. et al., 2002; Liu et al., 2022; Mekmene and Gaucheron, 2011). Parker & Dalgleish. (1981) reported lower calcium binding for β-casein than for α_s1_-casein which they related to the lower number of phosphoserine residues in β-casein. Differences are also apparent in the calcium sensitivity of the αs-caseins. Aoki et al. (1985) reported that approximately 94% of α_s2_-casein precipitated at 2 mM CaCl_2_, whereas α_s1_-casein showed almost no precipitation under the same conditions (0.1% protein, pH 7). Their dephosphorylation experiments linked the high calcium sensitivity of α_s2_-casein to its phosphate groups (Aoki et al., 1985). For α_s1_-casein, precipitation has been reported above approximately 6.5 mM Ca^2+^ at pH 7 and 20 °C in 0.02 M imidazole/HCl buffer at 0.05% protein (Horne and Dalgleish, 1979). The proposed mechanisms involve Ca^2+^-mediated neutralization of negatively charged groups, including phosphate groups, which reduces electrostatic repulsion and promotes protein–protein interactions and precipitation (Aoki et al., 1985; Horne and Dalgleish, 1979). Overall, casein fractions are commonly classified according to their calcium sensitivity, in which α_s1_-, α_s2_-and β-casein are calcium-sensitive, whereas κ-casein is calcium-insensitive (Müller-Buschbaum et al., 2007). Calcium insensitivity, however, does not imply an absence of calcium binding. Fitzgerald & Swaisgood (1989) identified a moderate-affinity Ca^2+^-binding site in κ-casein with a dissociation constant of 15.6 μM and proposed that the site involved a negatively charged region that probably included its phosphate group. The effect of phosphorylation on calcium-dependent behavior was again observed by Chezan et al. (2026), in which phosphorylated β-casein showed lower solubility and greater aggregation at elevated calcium concentrations than non-phosphorylated recombinant β-casein. Calcium binding is also temperature dependent. Liu et al. (2022) showed that both calcium-binding capacity and affinity of commercial αs-casein preparation increased between 25 and 50 °C. For β-casein, calcium additionally produces ion-specific effects on aggregation. Li et al. (2019)reported substantially larger β-casein aggregates in the presence of Ca^2+^ than Mg^2+^. Magnesium ions induced the formation of relatively small aggregates (≈56 nm), whereas calcium ions promoted much larger aggregates (≈450 nm) (Li et al., 2019). Calcium was also shown to promote β-casein aggregation through two key mechanisms: (1) Ca^2+^-mediated bridging between β-casein molecules and (2) reduction of electrostatic repulsion via ion binding and charge screening. Consistent with this, the addition of 30 mM CaCl_2_ at pH 7, shifted the zeta potential from −44.4 mV to −16.5 mV accompanied by an increase in particle size (McCarthy et al., 2014). These calcium-dependent effects also extend to β-casein at oil-water interfaces. Calcium has been reported to destabilize β-casein-stabilized emulsions (Dauphas et al., 2008). Notably, β-casein emulsions flocculated after 20 min at 55 °C in the presence of calcium, showing significantly altered physical properties compared with β-casein alone or β-casein/Mg systems across the investigated temperature range of 5–55 °C (Li et al., 2020). Calcium can alter β-casein interfacial properties that are directly relevant to foaming. At Ca^2+^:β-casein ratios of approximately 5:1 and above, Vessely et al. (2005) observed faster interfacial gelation, increased interfacial viscoelasticity, and a shift from interfacial aggregates toward a less ordered, more uniform protein layer (Vessely et al., 2005). Although calcium binding by caseins has been widely studied, direct quantitative comparisons between individual fractions remain limited because reported values depend strongly on protein purity, phosphorylation state and experimental conditions. The available evidence nevertheless demonstrates clear fraction-specific differences, particularly in calcium sensitivity, while also showing that phosphorylation and environmental conditions can strongly modify calcium-dependent behavior.

### 2.6 Solubility

The solubility of individual casein fractions results from a complex interplay of protein-solvent and protein-protein interactions, influenced by intrinsic molecular characteristics and environmental conditions such as pH, temperature, ionic strength and mineral composition (Huppertz et al., 2018). Near the pI, reduced net charge and electrostatic repulsion can allow for intermolecular association and precipitation. The distinct pI values of the individual casein fractions are one of the reasons for differing solubility profiles across pH. Sequence-based calculations predicted pI values of 4.91, 8.34, 5.13 and 5.93 for non-phosphorylated α_s1_-, α_s2_-, β- and κ-caseins, respectively. For the corresponding phosphorylated reference variants (α_s1_-casein B-8P, α_s2_-casein A-11P, β-casein A2-5P and κ-casein A-1P), the predicted pI values decreased to 4.42, 4.95, 4.65 and 5.60, respectively (Huppertz et al., 2018). This shows that phosphorylation alters casein charge. Consistent with the importance of charge and pH, they generally have reduced solubility near their pI, although their response to temperature differs markedly. β-casein is the most temperature-sensitive, showing increased solubility at lower temperatures (most notably between 5 °C and 15 °C) across pH 3.9-5.2. κ-casein had a narrower temperature response; solubility increased with decreasing temperature only between pH 4.7 and 5.2, with little effect observed from 3.9 to 4.6. α_s1_-casein was the least temperature-sensitive, and only marginally more soluble at 5 °C than at 25 °C, showing minimal variation across the tested pH and temperature ranges. These findings highlight distinct solubility behaviors likely linked to structural differences, although the narrow pH range studied is a limitation (Bingham, 1971). Later work by Post et al. (2012) further showed that αs-casein solubility is less affected by temperature across a broad pH range (2–11), whereas β-casein displays a much stronger temperature dependence, with increased solubility at lower temperatures, particularly near its pI. PTMs further modulate these differences by altering charge and mineral-binding behavior.

## 3 Food-relevant functional properties of casein fractions

The distinct physicochemical properties of casein fractions significantly influence the texture, structure, and sensory attributes of food products. A comprehensive understanding of these properties is essential for their targeted application and optimization in the formulation of dairy products.

### 3.1 Gelation ability

There have been few studies on the gelation of individual casein fractions, with β-casein being the most extensively investigated. For instance, a 2% β-casein solution acidified with 0.25% GDL formed a weak acid-induced gel (G′ ≈ 1 Pa). Interestingly, gelation was absent in partially dephosphorylated β-casein (McCarthy et al., 2013). However, Chezan et al. (2026) demonstrated that β-caseins (i.e., precision fermentation-derived, native bovine-derived phosphorylated and dephosphorylated) can form acid-induced gels. This finding further confirms that acid-induced gelation can occur outside the native casein micelle structure. The gelation behavior of the three differed, with recombinant and dephosphorylated native β-caseins forming a gel faster at a higher pH and forming stronger networks than phosphorylated native β-casein. These differences may be attributed to a reduced degree of phosphorylation, which increases the pI and promotes gelation at a higher pH where electrostatic repulsion is minimized. The observed gels were weak, with a maximum G′ of ≈9.26 Pa. Together, these findings indicate that acid-induced gelation of isolated β-casein depends strongly on phosphorylation state and experimental conditions. Differences in protein concentration and solution conditions may therefore contribute to the contrasting gelation behavior reported by McCarthy et al. (2013) and Chezan et al. (2026).

Although this process differs mechanistically from slow GDL-induced gelation, β-casein was also able to form gels at pH below 5.7. Gelation was observed at a concentration of 4.5% in pure water and was enhanced by elevated temperatures and the addition of sodium polyphosphate. Notably, sodium polyphosphate enabled gelation at concentrations as low as 0.4% at 20 °C, likely by facilitating interactions between positively charged sites on the β-casein micelles. Additionally, gel strength increased at lower temperatures, suggesting that hydrogen bonding may play a role in the gel network formation (Panouillé et al., 2005).

Neither β-casein nor αs-casein formed gels under neutral-to-alkaline conditions and high concentrations in the following studies. At a concentration of 6%, β-casein (≥98% purity) failed to form a gel in 0.2 M NaCl at pH 7.8 and ambient temperature (Yuno-Ohta and Corredig, 2011). Similarly, αs-casein (purity >70%) failed to form a gel at a concentration of 12% after being heated to 80 °C for 20 min at pH 7 in a 10 mM sodium phosphate buffer solution (Matsudomi et al., 2003). These findings indicate that neutral-to-alkaline conditions inhibit gelation in both casein fractions under the specific conditions investigated. Although no studies on gelation of intact κ-casein protein were found, two studies have specifically investigated the gelation behavior of its derived peptide, casein glycomacropeptide (CMP) (Martín-Diana et al., 2006; Martinez et al., 2011). CMP is a hydrophilic fragment of κ-casein that is released during enzymatic cleavage at the Phe105-Met106 bond. CMP can undergo pH-dependent self-assembly at room temperature, gradually forming various supramolecular structures in the process. At pH values below 4.5, CMP formed opaque, elastic gels at concentrations as low as 3% (w/w). While this gelation is strongly pH-dependent, it can be modulated by temperature; heating up to 70 °C accelerated gelation and enhanced gel strength, particularly at pH values between 2.0 and 3.5. The resulting gels were partially reversible with respect to pH changes, mainly reverting to dimeric forms (Martinez et al., 2011). In contrast, Martín-Diana et al. (2006) demonstrated that CMP can form gels at neutral pH, but only at a relatively high protein concentration of around 20% (w/w). Gelation occurred at moderate temperatures, with an onset at 44 °C. Although these findings cannot be extrapolated directly to intact κ-casein, they demonstrate distinct gel-forming behavior of its CMP-derived region under appropriate conditions. Beyond their ability to form protein gels independently, isolated casein fractions can also modify the structure of composite gel systems through interactions with polysaccharides and other proteins. One study investigated the effect of individual caseins (α-, β- and κ-casein, each ≥90% pure) on the rheology of ι-carrageenan gels. Controlled stress oscillatory rheology was used on 1% ι-carrageenan gels (with CaCl_2_ or KCl) to show that κ-casein had the most pronounced effect, significantly increasing gel rigidity. β-casein also increased stiffness, albeit to a lesser extent than κ-casein, while α-casein had the least impact. These results suggest that the different casein fractions interact distinctly with ι-carrageenan, with κ-casein being the most effective at reinforcing the gel network (Lynch and Mulvihill, 1994). When combined with 6% β-Lactoglobulin (BLG) and sodium caprate in 0.2 M NaCl at a pH of 7.8 and at ambient temperature, β-casein behaved as a texture modifier in a system that was not acid-induced (Yuno-Ohta and Corredig, 2011). Similarly, the addition of calcium ions at pH 7.0 and NaCl concentrations of up to 80 mM enhanced gelation in mixtures with κ-carrageenan by promoting cross-linking between the polymers. Under these conditions, β-casein promoted the formation of a stronger ion-mediated gel network (Ozawa et al., 1985). In conclusion, few studies have examined the gelation behavior of individual casein fractions in detail, and the available evidence focuses mostly on β-casein. Although casein micelles are well known to form gels through different routes (i.e., acidification, enzymatic action, heat, or salt), the individual casein fractions appear to have more limited gelation mechanisms. Isolated β-casein can form acid-induced gels without native micellar organization, although these gels are generally weak and strongly dependent on phosphorylation state, protein concentration and solution conditions. This may be linked to the lack of micellar structure, which supports multiple interactions in gelation. In contrast, direct evidence for independent gel formation by isolated α_s1_-, α_s2_-and intact κ-casein remains very limited. CMP exhibits distinct gel-forming behavior, but these findings should not be considered equivalent to gelation of intact κ-casein. Individual fractions may also modify gel structure in composite protein-polysaccharide systems. The limited fraction-specific evidence may be more a reflection of practical issues than a lack of scientific interest.

### 3.2 Emulsifying ability

The emulsifying property of a protein refers to its ability to migrate to the oil-water interface, reduce interfacial tension and form a stabilizing layer. The effectiveness with which proteins perform these functions depends on intrinsic characteristics like amino acid sequence and structure, as well as protein concentration, and environmental factors, including pH, ionic strength, temperature and the nature of the oil phase (Chen et al., 2022). Evidence from micellar systems also indicates a high interfacial activity of β-casein. Zhou et al. (2022) showed that it was small casein aggregates and β-casein, rather than intact micelles, that dominated the oil-water interface. This suggests that under the conditions used, β-casein contributed substantially to the interfacial activity. The high interfacial activity of β-casein is further supported by a study of highly purified casein fractions outside the micellar structure. β-casein reduced surface tension faster than all other fractions, both in the presence and absence of simulated milk ultrafiltrate (SMUF) containing calcium and salts (De Groot et al., 2025). α_s1_-and β-caseins have noteworthy emulsifying properties due to their limited secondary structure and their amphiphilic nature (Cayot et al., 1991). However, when directly compared, differences were observed between α_s1_-and β-caseins in oil-in-water (O/W) emulsions with a low protein-to-fat ratio (1:20) and a pH range of 5.5-7. β-casein showed strong resistance to flocculation at low to medium ionic strengths (0.01-0.2 M) and near its pI, suggesting that its stabilization mechanism is primarily steric rather than electrostatic. In contrast, α_s1_-casein-stabilized emulsions began to flocculate at ionic strengths above 0.1 M and when the pH dropped below 5.5. This reduced ability to prevent flocculation is likely due to different interfacial layer structures and less effective steric stabilization under the measured conditions (Dickinson et al., 1998). These experimental results were supported by the theoretical work of Dickinson (1999), where the structure and interactions of α_s1_-and β-casein layers adsorbed at hydrophobic surfaces were predicted using the Scheutjens–Fleer self-consistent field (SCF) theory. The results, which are directly relevant to emulsifying behavior, showed that β-casein forms a relatively thick, two-layer structure at the interface with greater extension into the aqueous phase and more complete surface coverage, particularly at lower pH. By contrast, α_s1_-casein was predicted to adsorb as a thinner, loop-dominated layer with limited extension away from the interface (Dickinson, 1999). The comparatively high interfacial activity of β-casein is also reflected in its ability to reduce interfacial tension. At pH 7.0, β-casein showed the highest surface activity, reducing oil-water interfacial tension most effectively (19 mN/m), followed by κ-casein (22 mN/m) and αs-casein (27 mN/m) (Li and Zhong, 2021). For β-casein, this interfacial behavior has also been related to its highly charged, hydrophilic N-terminal domain. Comparative studies using β-casein peptide fragments have shown that the N-terminal fragment (β-casein f1–105/107) has superior emulsion-stabilizing properties to an internal fragment (β-casein f29–105/107). The adsorption of the N-terminal region at the oil-water interface may be more effective due to its balance of hydrophilic and hydrophobic residues (Caessens et al., 1999). While these studies demonstrate the high interfacial activity of β-casein, efficient adsorption does not necessarily ensure long-term emulsion stability. Emulsions prepared with three purified fractions (i.e., α_s1_-, β- and κ-caseins) at a low protein-to-fat ratio of 1:100 showed creaming after 28 days, indicating limited long-term stability. Microstructurally, α_s1_-casein promoted droplet aggregates, while β- and κ-caseins adsorbed more uniformly at the interface. Accordingly, emulsions stabilized by α_s1_-casein were less stable than those stabilized by β- and κ-caseins under the conditions studied. Unlike the previously studied system by Dickinson et al. (1998), which used a 1:20 protein-to-fat ratio, this system had fivefold less protein relative to fat content, which may have resulted in insufficient protein coverage and contributed to the observed instability (Li and Zhong, 2021). However, the stabilizing effect of β-casein is concentration- and system-dependent. When β-casein remains unadsorbed in the continuous phase, it can induce depletion attraction between emulsion droplets. Increasing β-casein concentration from 0.1 to 0.5% (w/w) strengthened depletion attraction between emulsion droplets (Dimitrova and Leal-Calderon, 2000). Furthermore, high β-casein concentrations do not necessarily result in destabilization, as their effect also depends on the composition of the continuous phase. β-casein concentrations of up to 10% (w/w) can enhance the stability of oil/water nano-emulsions (pH 7.5 at 25 °C) in the presence of lactose and trehalose. In systems with approximately 7.5-10% β-casein and 10-12.5% lactose or trehalose in the aqueous phase, with 20% (w/w) total solids and 10% (w/w) sunflower oil, both sugars synergistically reduced viscosity and inhibited flocculation. The most stable emulsions occurred at 7.5-10% β-casein, highlighting the dependence of β-casein stabilization on the composition of the continuous phase (Maher et al., 2011). Besides concentration and formulation, preparation conditions, particularly the order of addition, also influence the interfacial behavior of β-casein. When added to a model system containing polar and amphiphilic milk lipids, β-casein formed a stable interfacial layer with small droplets and resisted displacement by surface-active lipids. In contrast, adding lipids first hindered protein adsorption, while simultaneous addition led to competition for adsorption at the oil–water interface (Waninge et al., 2005). Similarly, interfacial studies of mixed protein systems (e.g., β-casein and bovine serum albumin (BSA)) demonstrated that adsorption dynamics and interfacial layer structure are strongly influenced by the sequence of adsorption (Chrysanthou et al., 2024). Concentration-dependent changes in interfacial composition have also been observed in mixed casein systems. In sodium caseinate emulsions, β-casein dominates interfacial adsorption at low protein concentrations (≤2%). For instance, with sodium caseinate (1% w/w protein, 20% oil), β-casein showed greater interfacial adsorption than other casein fractions (Brun and Dalgleish, 1999). At higher concentrations, however, αs-caseins increasingly adsorbed and eventually dominated the interface, indicating a concentration-dependent shift in interfacial composition (Chen et al., 2022; Euston et al., 1996; Srinivasan et al., 1996; Ye, 2008). This shift has been attributed to β-casein's strong tendency to self-aggregate and form micelles at higher concentrations (>2%), which might limit its ability to adsorb at the interface (Berot et al., 2003; Euston et al., 1996; Srinivasan et al., 1996). While direct comparisons among the casein fractions remain limited, comparisons with globular proteins provide additional insight into the interfacial behavior of β-casein. Cheetangdee et al. (2011) and Berton et al. (2011) investigated the interfacial behavior of β-casein, BLG and BSA at 25 °C in 10 mM buffers. Berton et al. (2011) showed that β-casein had the highest initial interfacial adsorption, with only 4.6–9.0% of the protein remaining unadsorbed, compared to 27.3–29.3% for BSA and 30.4–32.8% for BLG. Cheetangdee et al. (2011) found that β-casein also reduced interfacial tension by 20 mN/m at a lower concentration of ≈0.07 g/L and emulsified up to 20% oil, which exceeds the emulsification capacity of both BLG (10%) and BSA (5%). However, both studies also noted that β-casein was less stable at the interface, as indicated by larger droplet sizes (around 20 μm), increased desorption over 48 h (up to 20%) and greater susceptibility to coalescence at pH 7. This is likely due to its disordered multilayer structure. In contrast, BLG and BSA formed smaller droplets and more stable interfacial films, with negligible desorption over time. These studies indicate that the rapid adsorption and high oil-binding capacity of β-casein do not necessarily translate into greater long-term emulsion stability. This difference may be related to the more rigid interfacial structures that are formed by globular proteins. This is further supported by a comparison of β-casein with the globular protein soy 11S globulin (glycinin), which forms more cohesive interfacial films that effectively resist coalescence (Berot et al., 2003). Overall, these findings further support that the diffuse, less cohesive structure of the β-casein interface contributes to its lower emulsion stability compared to that of more structured globular proteins (Berot et al., 2003; Chrysanthou et al., 2024; Dickinson, 1999). However, when focusing on lipid–protein interaction strength rather than adsorption behavior, a different trend emerges. Aynié et al. (1992), showed that, in a model system using stearic acid homologues, interactions followed the order α_s1_-casein > β-lactoglobulin > β-casein, with α_s1_-casein associated with greater interfacial organization, likely due to stronger electrostatic and hydrogen-bonding interactions related to its higher net positive charge. The dynamic nature of the β-casein interface can also be seen under competitive adsorption conditions. When used as a purified protein, β-casein can be displaced from the oil–water interface by nonionic surfactants (e.g., Tween 20 and octaethylene glycol monododecyl ether) and phospholipids (e.g., lecithin). In contrast, more interfacially robust proteins, such as BLG and BSA, resist displacement and form more stable films. This indicates that β-casein adsorption is highly dynamic and that its interfacial film is comparatively weak under competitive surfactant conditions, which can negatively affect emulsion stability (Berton-Carabin et al., 2013; Cayot et al., 1991; Chen and Dickinson, 1993; Cornec et al., 1996; De Feijter et al., 1987). This interpretation is further supported by interfacial shear rheology studies, which show that although β-casein can dominate surface adsorption, it forms comparatively soft and less mechanically robust interfacial networks than BSA, potentially contributing to its susceptibility to displacement under competitive conditions (Chrysanthou et al., 2024). In general, β-casein shows particularly high interfacial activity among the casein fractions, whereas α_s1_-casein also emulsifies but is more sensitive at low pH (<5.5) and high ionic strength (>0.1 M) under the conditions studied. Available direct comparisons indicate that κ-casein can also adsorb effectively at the oil–water interface, although considerably less fraction-specific evidence is available than for β-casein. In contrast, evidence on the emulsifying behavior of isolated α_s2_-casein is particularly limited.

### 3.3 Foaming properties

Foamability refers to proteins' ability to promote foam formation by adsorbing at the air–water interface. Upon adsorption, proteins can rearrange to form viscoelastic interfacial films that stabilize gas bubbles against coalescence and disproportionation. This functionality is important for the texture and stability of aerated dairy products such as whipped cream and milk foams (Narsimhan and Xiang, 2018). Foaming behavior can be characterized through different parameters, including adsorption kinetics, surface tension, interfacial rheology and film properties, as well as macroscopic measures of foam formation and stability. Consequently, results obtained using different experimental approaches are not always directly comparable. A notable study by De Groot et al. (2024) evaluated the interfacial properties of all four major casein fractions (α_s1_-, α_s2_-, β- and κ-casein) using oscillating bubble tensiometry in simulated milk ultrafiltrate containing Ca^2+^. β-Casein formed the stiffest interfacial network, with a network contribution of approximately 30 mN/m at low strain and 19 mN/m at high strain, followed by α_s2_-and α_s1_-casein. κ-casein, in contrast, produced the weakest network, at approximately 11 mN/m, with little dependence on strain. In milk-like blends, β- and κ-casein dominated the interfacial response, and the presence of Ca^2+^ was essential for formation of a stiff network (De Groot et al., 2024). Recently, Mortes et al. (2026) found similar foamability but different foam stability for recombinant α_s1_-casein variants A and B. The more mobile interface formed by variant B permitted Gibbs–Marangoni flow and greater stability at low concentration, showing that genetic variation can alter foam-stabilization mechanisms. Furthermore, β- and α_s1_-caseins showed similar adsorption behavior when studied individually at 24 °C in 20 mM phosphate buffer at pH 7, reaching saturation at approximately 1.7–1.8 mg/m^2^ above a bulk concentration of 1.5 mg/mL. However, in a 1:1 mixture, β-casein became dominant at the interface, displacing α_s1_-casein over time and establishing an equilibrium surface ratio of approximately 2:1. This behavior was associated with the slightly higher binding constant of β-casein (4.0 × 10^4^ vs 3.6 × 10^4^ mg/(m^2^·wt%)) and its greater mean residue hydrophobicity (1330 vs 1170 cal/mol). These results indicate that relatively small differences in hydrophobicity and surface affinity can substantially influence competitive adsorption and interfacial behavior (Anand and Damodaran, 1996). Casein concentrates with a higher proportion of β-casein demonstrate improved foam stability and emulsifying capacity, primarily due to the hydrophobic nature of β-casein (Murphy and Fox, 1991; Seibel et al., 2015). This trend also applies to camel casein, which has a higher proportion of β-casein than bovine casein, highlighting the important roles played by both β-casein content and hydrophobicity in improving the interfacial properties of mixed casein systems (Lajnaf et al., 2021, 2022).

Although Barackov et al. (2012) investigated β-casein in a foam-fractionation process rather than in a food foam, the study provides useful information on its gas-liquid interfacial behavior. Across the tested range of pH 5.9–8.7, ionic strength 0.03–0.17 M and 23–37 °C, β-casein enrichment increased with pH and was highest at pH 8.7, 0.1 M ionic strength and 30 °C. Higher ionic strength at elevated pH reduced enrichment, which the authors attributed to charge screening and decreased protein solubility. Under the conditions giving maximum enrichment, β-casein adsorbed rapidly and mainly reoriented at the interface, with no pronounced unfolding or irreversible aggregation (Barackov et al., 2012). The association state of β-casein also influences its interfacial adsorption. In a spray-drying system, Landström et al. (2003) found that monomeric β-casein showed higher affinity for the air–water interface than Ca^2+^-induced associated β-casein, indicating that calcium-induced association can reduce its interfacial adsorption (Landström et al., 2003). Foaming ability is not entirely determined by interfacial affinity. Thus, when considering direct foaming in bulk solutions, higher concentrations of β-casein can improve foaming stability. For instance, samples containing 0.015% and 0.15% β-caseins by weight both achieved a final foam volume of 120 ml after 40 s of sparging and had strong foamability across this concentration range. Moreover, foam stability, as indicated by the foam half-life, increased significantly with concentration from 260 ± 20 min at 0.015% to 455 ± 7 min at 0.15%. These findings suggest that increasing β-casein concentration in the bulk solution contributes to foam stability (Chen et al., 2018). Concentration also affected foam formation: Martin et al. (2002) found that, for β-casein, foam volume increased with concentration up to 0.3 g/L (0.03% w/v), but did not increase further at higher concentrations under the experimental conditions.

Several studies have shown that a minimum concentration of β-casein is required to produce consistent foams in phosphate buffer systems. Using sparging, Ahmed & Dickinson (1991) found that a concentration of 0.005% (w/w) was insufficient to generate stable foam at neutral pH and room temperature. In contrast, Bos et al. (2003) and Burke et al. (2014) used manual shaking to produce foam, but obtained different results. In Burke et al. (2014), 0.1% (w/w) of β-casein failed to produce a stable foam. Conversely, Bos et al. reported that a concentration of around 0.14% (w/w) was sufficient for consistent foam formation under similar conditions, such as manual shaking and near neutral pH. The ability of β-casein to stabilize foams at increasing concentrations can be explained by its dynamic behavior at the air-water interface. It remains fluid through the desorption of its hydrophilic regions into the subphase. This process enables molecular mobility even at higher surface concentrations above 2.6 mg/m^2^, thereby supporting stable foam formation (Cicuta, 2007).

The interfacial conformation of β-casein depends on pH and surface pressure. Below 15.4 mN/m at pH 5 and 10.5 mN/m at pH 7, the protein adopts a tail-train structure. As surface pressure increases due to interfacial crowding, β-casein transitions to a loops-and-tails structure, in which segments protrude into the subphase. This transition reflects the protein's intrinsically disordered structure and its ability to adapt to packing constraints, with pH modulating the pressure threshold for conformational rearrangement (Caro et al., 2009). Even dissolved at a pH of 3.7, 10 mM citrate buffer, at room temperature and a concentration of 0.1%, where both β-casein and sodium caseinate carry a net positive charge, β-casein had superior foamability (1.18 ± 0.04 versus 0.85 ± 0.07). It is noted that the foamability was measured by the initial foam volume relative to the initial liquid volume. Moreover, this result can be attributed to the flexible and amphiphilic structure of β-casein, which facilitates foaming despite reduced electrostatic repulsion. Conversely, sodium caseinate produced more stable foams over 3 h (78 ± 11% vs 60 ± 1%), indicating a trade-off between foam formation and stability. One limitation of the study is the use of a manual shaking method for 30 s, which may introduce variability (Yucel Falco et al., 2017). Therefore, foam formation and foam stability can respond differently to protein composition.

In a comparative study of intact β-casein and its plasmin-derived peptides, Caessens et al. (1997) found that fractions enriched in hydrophobic C-terminal peptides showed improved foam-forming and foam-stabilizing properties compared with intact β-casein, particularly at pH 4.0. In contrast, the hydrophilic N-terminal peptide fraction showed the opposite properties.

Differences between casein fractions are also evident in their adsorption behavior. At 0.001% (w/w), β-casein and sodium caseinate reduced surface tension to 55 ± 1 and 54 ± 1 mN/m, respectively, whereas κ-casein reached 61.3 ± 0.2 mN/m; at the same concentration, κ-casein also adsorbed more slowly than β-casein (Ridout et al., 2015). Time-dependent changes also affect β-casein interfacial layers. At β-casein concentrations above 0.001% (w/w), the interfacial layer thickened over approximately 10–15 h and developed a more rigid, gel-like structure. These observations indicate that the mechanical properties of β-casein films evolve progressively after adsorption. (Bantchev and Schwartz, 2004). The adsorption of β-casein is also sensitive to solution conditions. Damodaran & Song (1990) found that 1 M NaCl increased both the rate and equilibrium adsorption of β-casein at the air–water interface, whereas 1 M NaSCN decreased both relative to phosphate buffer. They attributed these opposing effects to the different effects of the kosmotropic and chaotropic salts on bulk water structure and, consequently, on the hydrophobic effect (Damodaran and Song, 1990). Processing can likewise modify foam performance. High-pressure treatment of β-casein solutions at 300 MPa improved foam stability and slowed film thinning, while treatment for 30 min also increased equilibrium film thickness. The treated samples were more resistant to destabilization by Tween 20. Although no substantial changes in hydrophobicity or secondary structure were detected, the molecular mechanism underlying these improvements remained unresolved (Pittia et al., 1996). The stability of foam from casein can also be enhanced with the addition of ethanol in the range of 0.2-0.3% (w/w), with β-casein outperforming lysozyme but remaining less stable than gelatin. The improved interfacial behavior of β-casein under controlled conditions (pH 7, 25 °C) may potentially be due to decreased polarity of the medium. However, the broader environmental effects were not explored. The order of foam stability among the tested proteins was as follows: gelatin > β-casein > BLG > lysozyme (Ahmed and Dickinson, 1990).

There is limited research into the individual foaming properties of highly purified α_s1_-, α_s2_-and intact κ-casein, since most studies use enriched or mixed casein systems. Some studies have instead examined casein glycomacropeptide (CMP), a chymosin-released fragment of κ-casein. Martinez et al. (2012) reported that 4% CMP showed high foaming capacity but poor foam stability, with complete foam collapse occurring after approximately 700 s. The CMP-stabilized interface also showed relatively low dilatational elasticity and viscosity, consistent with its limited resistance to foam destabilization (Martinez et al., 2012). Glycosylation further influenced CMP functionality. Kreuß et al. (2009) found that glycosylated and non-glycosylated CMP had similarly high foamability, but glycosylation markedly reduced foam rigidity and stability, particularly away from the pI (Kreuß et al., 2009). CMP should, however, not be considered equivalent to intact κ-casein.

Protein structure also influences adsorption and foam formation. Globular and disordered proteins show distinct interfacial behavior at the air–water interface. Rodríguez Patino et al. (2001) compared β-casein, caseinate and whey protein isolate (WPI) monolayers at pH 5 and 7 and found that their dilatational properties depended on protein type, pH and interfacial concentration. In β-casein films, interfacial packing was more compact near the pI (pH ~ 5), whereas at pH 7 the films were less condensed. The dilatational properties also varied with surface concentration, indicating that the mechanical response of β-casein monolayers depends strongly on both pH and interfacial packing (Rodríguez Patino et al., 2001).

When compared to gliadin and glycinin, β-casein produced the highest amount of foam at a concentration of 1.4 g/L (0.14% w/v). This superior foaming ability is largely due to the faster adsorption rate of β-casein at the air-water interface compared to the slower adsorption rate of plant proteins. Therefore, adsorption kinetics and molecular flexibility are both key factors affecting foam formation and stability (Bos et al., 2003). High foamability does not necessarily imply the formation of a mechanically strong interfacial film. Martin et al. (2002) further demonstrated the importance of adsorption kinetics, showing that although β-casein had the weakest network-forming ability compared with BLG, ovalbumin, and soy glycinin, it was the most effective foaming agent. Their results indicated that rapid adsorption and conformational rearrangement are particularly important for foam formation, whereas stronger interfacial networks can favor subsequent foam stability. Furthermore, compared to BSA and lysozyme, β-casein demonstrated superior foamability, even at markedly lower concentrations (0.015% for β-casein, 0.05% for BSA and 3% for lysozyme) (Dickinson and Izgi, 1996).

Although β-casein generally performs well in foam formation, it showed poor stability against disproportionation compared with hydrophobin HFBII, quillaja saponin and BLG at 0.1%. Coarsening began immediately after foam formation, and β-casein-stabilized foams collapsed first. This rapid coarsening was associated with the relatively low mechanical resistance of the β-casein interfacial layer (Blijdenstein et al., 2010). Low-molecular-weight surfactants can also disrupt protein films. Clark et al. (1994) showed that competitive adsorption of Tween 20 caused breakdown of protein–protein interactions and partial displacement of adsorbed protein from β-casein-containing interfaces (Clark et al., 1994). Conversely, β-casein can have synergistic effects with other surface-active compounds. Of several proteins tested, including BLG, gelatin and soy protein, β-casein demonstrated the strongest synergistic interaction with propylene glycol alginate (PGA) at pH 7. This resulted in the most stable and voluminous foams. The enhancement is attributed to favorable interactions at the air-water interface, likely driven by weak electrostatic complexation between β-casein and PGA. Foam volume was greatest near the β-casein pI (pH ~ 5.2–5.3), consistent with enhanced interfacial film strength near the isoelectric point (Ahmed and Dickinson, 1991). Other synergistic effects involving β-casein have been reported. For example, when combined with HFBII (hydrophobin II), an increase in surface shear viscosity was observed (Burke et al., 2014). Similarly, β-casein showed synergistic interactions with Quillaja bark saponin at low saponin-to-protein ratios, where the rate of interfacial tension reduction increased and equilibrium interfacial tension decreased; however, the effect depended on mixture composition and the QBS preparation used (Kezwon and Wojciechowski, 2014). Similarly, CMP also exhibited synergistic interfacial effects when combined with PGA (78–85% highly esterified propylene glycol alginate). This interaction increased the dilatational elasticity and viscosity of the film and improved foam stability against drainage and initial collapse (Martinez et al., 2012).

### 3.4 Degradation of casein fractions

Caseins are highly susceptible to degradation, which results in the formation of smaller peptides and free amino acids. This process can occur naturally or be catalyzed by enzymes like chymosin or microbial proteases. Proteolysis occurs during milk storage, processing, fermentation and cheese ripening. The majority of naturally occurring peptides in raw cow's milk result from casein proteolysis. Of the 248 peptides identified, almost all originated from casein fractions, with α_s1_-casein being the main source, followed by β- and α_s2_-caseins. Only a small proportion of peptides were derived from κ-casein and whey proteins (Baum et al., 2013). Proteolytic activity is primarily due to endogenous milk enzymes, plasmin and cathepsins. Casein-based peptides also show a strong structure-function relationship. Enzymatic hydrolysis can alter their food-related functionalities, such as solubility, emulsifying capacity, and foaming ability. Limited hydrolysis can enhance emulsification and modulate foaming. Hydrolysis reduces the size of the peptides, thereby exposing functional groups and influencing these properties. Therefore, the type of enzyme used, and the hydrolysis conditions are critical, as is maintaining the size of the peptides and their structural features. The clustering of hydrophilic and hydrophobic groups of the resulting peptide is also important (Darewicz et al., 2006). Evidence from complex dairy systems further demonstrates the sensory consequences of casein proteolysis. Proteolysis is a major contributor to the sensory characteristics of ripened cheeses such as Camembert. During ripening, indigenous milk enzymes and starter and surface-ripening cultures hydrolyze caseins, releasing peptides and free amino acids that influence flavor development. Bitterness is particularly associated with hydrophobic peptides, many of which are derived from β-casein (Kuhfeld et al., 2024). Other flavor attributes may also arise from casein breakdown, although they cannot always be linked to specific casein fractions. During ripening, casein proteolysis releases glutamate and glutamine into the free amino acid pool. Glutamate can contribute directly to umami taste, while these amino acids can also serve as precursors for taste-active γ-glutamyl peptides, such as γ-Glu-Leu and γ-Glu-Val, which are associated with kokumi-related attributes (Braitmaier et al., 2025). The profile of free amino acids is also influenced by the genotype of casein fractions. In particular, variations in α_s1_-κ-casein genotypes have been shown to affect sensory properties, with certain genotypes associated with higher sweetness and lower hardness, highlighting their role in modulating cheese texture and flavor (Olsen et al., 2023). The targeted use of individual casein fractions offers potential as functional food ingredients due to their distinct degradation patterns and ability to generate peptides with specific properties. Unlike intact casein micelles, isolated fractions may enable greater control over enzymatic hydrolysis, peptide generation and the final functional outcome. However, direct comparisons of proteolysis and the resulting functionality among purified individual casein fractions remain limited.

## 4 Current challenges and future directions

The level of knowledge varies across casein fractions and functional properties, making direct comparisons between the fractions difficult. Although individual casein fractions have been studied for decades, relatively few studies directly compare purified fractions under equivalent conditions. β-casein has been investigated considerably more extensively than the other fractions. Most studies reviewed here investigated fractionated native bovine caseins, but genetic variants were not consistently reported. Differences in purity, PTMs (i.e., phosphorylation and glycosylation), and experimental conditions further complicate establishing structure–function relationships. Developments in casein fractionation and precision fermentation provide two complementary directions for future research. For milk-derived fractions, consistent characterization of purity, genetic variant and PTM state would allow their functional properties to be more reliably compared. For recombinant caseins as derived from advances in biotechnology, future research could take a more function-specific approach, systematically investigating which molecular characteristics, including genetic variants and PTMs, are required for a defined functionality. This should also include determining whether individual fractions can provide the desired functional properties directly or whether their reassembly into more complex structures, including micelle-like structures, is required. We further note that yield and, subsequently, economic feasibility may also be important factors. Although these aspects are beyond the scope of the current review, they may be relevant when considering the use of casein fractions, either derived from fractionation or produced through biotechnology, in real-world food production applications.

## 5 Conclusion

Most of the literature reviewed here investigated bovine-derived casein fractions, while only a small number of studies examined recombinant caseins. Among the individual fractions, β-casein was the most extensively studied and was characterized by pronounced temperature-dependent self-association, high interfacial activity and the ability to form weak acid-induced gels. α_s1_-casein also showed self-association, calcium sensitivity and interfacial activity, although fewer studies have investigated its food-relevant functional properties compared to β-casein. The most distinctive property identified for α_s2_-casein was its particularly high calcium sensitivity, while evidence concerning its behavior in isolation remains scarce. κ-casein differs by its calcium insensitivity and its aggregation and self-assembly behavior, which can be influenced by disulfide bonding and glycosylation. Table 2 summarizes the key physico-chemical and food-relevant functional properties of the individual casein fractions identified from the literature reviewed here.

**Table 2:** Key properties of bovine casein fractions relevant to functional behavior in food systems.

Property	α_s1_-casein	α_s2_-casein	β-casein	κ-casein
Calcium binding/sensitivity	Calcium-sensitive	Particularly high calcium-sensitivity	Calcium sensitive	Considered calcium-insensitive
Aggregation behavior	Self-association reported	Very limited data in isolation	Self-association, dependent on temperature, pH, and ionic strength	Forms aggregates and is influenced by pH, temperature, and disulfide bonds
Gelation (isolated fractions)	Insufficient fraction-specific evidence	Insufficient fraction-specific evidence	Weak gels and dependent on pI and phosphorylation	Not reported for intact protein; CMP forms gels
Interfacial behavior oil/water	Emulsifying activity; stability sensitive to pH and ionic strength	Insufficient fraction-specific evidence	High interfacial activity and dynamic adsorption; susceptible to competitive displacement	Interfacial activity demonstrated in direct fraction comparison
Interfacial behavior air/water	Limited data; variant-dependent foam stability.	Interfacial activity demonstrated; limited direct foam data.	High foamability; stability is system dependent.	Limited data on intact fraction; CMP shows high foamability with condition-dependent stability
Research coverage	Moderate	Very limited	Most studied	Limited

Note: This table summarizes the functional properties of bovine casein fractions based on the literature reviewed in this study. The amount and strength of the available evidence differ among fractions, reflecting the current state of research.

## Data availability

The datasets can be made available by the corresponding author upon reasonable request.

## Declaration of generative AI and AI-assisted technologies in the manuscript preparation process

During the preparation of this manuscript, the authors used ChatGPT (OpenAI) to improve the language and readability of the text. Having used this tool, the authors reviewed and edited the content as required, taking full responsibility for the content of the published article.

## Declaration of competing interest

The authors declare that they have no known competing financial interests or personal relationships that could have appeared to influence the work reported in this paper.
